# Widespread grey matter pathology dominates the longitudinal cerebral MRI and clinical landscape of amyotrophic lateral sclerosis

**DOI:** 10.1093/brain/awu162

**Published:** 2014-06-20

**Authors:** Ricarda A. L. Menke, Sonja Körner, Nicola Filippini, Gwenaëlle Douaud, Steven Knight, Kevin Talbot, Martin R. Turner

**Affiliations:** 1 Oxford Centre for Functional Magnetic Resonance of the Brain, University of Oxford, UK; 2 Nuffield Department of Clinical Neurosciences, University of Oxford, UK; 3 Department of Neurology, Hannover Medical School, Germany; 4 Department of Psychiatry, University of Oxford, UK; 5 Oxford Centre for Magnetic Resonance Imaging Research, University of Oxford, UK

**Keywords:** motor neuron disease, biomarker, magnetic resonance imaging, voxel-based morphometry, diffusion tensor imaging

## Abstract

Diagnosis, stratification and monitoring of disease progression in amyotrophic lateral sclerosis currently rely on clinical history and examination. The phenotypic heterogeneity of amyotrophic lateral sclerosis, including extramotor cognitive impairments is now well recognized. Candidate biomarkers have shown variable sensitivity and specificity, and studies have been mainly undertaken only cross-sectionally. Sixty patients with sporadic amyotrophic lateral sclerosis (without a family history of amyotrophic lateral sclerosis or dementia) underwent baseline multimodal magnetic resonance imaging at 3 T. Grey matter pathology was identified through analysis of T_1_-weighted images using voxel-based morphometry. White matter pathology was assessed using tract-based spatial statistics analysis of indices derived from diffusion tensor imaging. Cross-sectional analyses included group comparison with a group of healthy controls (*n = *36) and correlations with clinical features, including regional disability, clinical upper motor neuron signs and cognitive impairment. Patients were offered 6-monthly follow-up MRI, and the last available scan was used for a separate longitudinal analysis (*n = *27). In cross-sectional study, the core signature of white matter pathology was confirmed within the corticospinal tract and callosal body, and linked strongly to clinical upper motor neuron burden, but also to limb disability subscore and progression rate. Localized grey matter abnormalities were detected in a topographically appropriate region of the left motor cortex in relation to bulbar disability, and in Broca’s area and its homologue in relation to verbal fluency. Longitudinal analysis revealed progressive and widespread changes in the grey matter, notably including the basal ganglia. In contrast there was limited white matter pathology progression, in keeping with a previously unrecognized limited change in individual clinical upper motor neuron scores, despite advancing disability. Although a consistent core white matter pathology was found cross-sectionally, grey matter pathology was dominant longitudinally, and included progression in clinically silent areas such as the basal ganglia, believed to reflect their wider cortical connectivity. Such changes were significant across a range of apparently sporadic patients rather than being a genotype-specific effect. It is also suggested that the upper motor neuron lesion in amyotrophic lateral sclerosis may be relatively constant during the established symptomatic period. These findings have implications for the development of effective diagnostic versus therapeutic monitoring magnetic resonance imaging biomarkers. Amyotrophic lateral sclerosis may be characterized initially by a predominantly white matter tract pathological signature, evolving as a widespread cortical network degeneration.

## Abstract (precis)

Menke/Koerner *et al.* use structural MRI to explore the extent of longitudinal changes in cerebral pathology in amyotrophic lateral sclerosis, and their relationship to clinical features. A characteristic white matter tract pathological signature is seen cross-sectionally, while cortical involvement dominates longitudinally. This has implications for the development of biomarkers for diagnosis versus therapeutic monitoring.

## Introduction

The neurodegenerative pathology in amyotrophic lateral sclerosis (ALS) is characterized by loss of upper motor neurons of the primary motor cortex and corticospinal tract, lower motor neurons of the brainstem and spinal cord; and extramotor pathology that has clinical, pathological and genetic overlap with frontotemporal dementia (Kiernan *et al.*, 2011). There is no highly effective disease-modifying treatment but, although the median survival is 3–4 years from symptom onset, there is considerable variation in progression rate overall. Upper and lower motor neuron involvement is found at post-mortem in nearly all cases with ALS, but upper motor neuron signs may be difficult to detect clinically (Swash, 2012). A very rare variant known as primary lateral sclerosis demonstrates only upper motor neuron involvement clinically, with characteristically long survival, and those with ‘pure’ lower motor neuron involvement are also typically slower in rate of progression (Turner *et al.*, 2003). Half of patients with ALS demonstrate evidence of frontotemporal cognitive impairment, independently linked to faster progression (Elamin *et al.*, 2011). ALS can thus be considered a syndrome, in which there is a variable extent of pathology within upper motor neuron, lower motor neuron and extramotor neuronal populations. Although genetic factors may influence the dominance of pathological involvement to some extent (Al-Chalabi *et al.*, 2012), 95% of patients report no family history of ALS or frontotemporal dementia, and currently have no obvious single genetic mutation that accounts for their disease despite indistinguishable clinical appearances.

This clinical heterogeneity, coupled with the insidious onset of symptoms in ALS, contributes to a mean interval of 1 year from symptom onset to diagnosis in ALS (Mitchell *et al.*, 2010). There are no fully validated biomarkers for diagnosis, stratification or therapeutic monitoring in ALS, although candidates are emerging from neuroimaging, body fluids and neurophysiology (Bowser *et al.*, 2011). Meanwhile, ALS remains reliant on clinical history examination for diagnosis, stratification and assessment of change, particularly in the setting of a therapeutic trial.

The brain appears normal on routine clinical MRI studies in ALS. Corticospinal tract hyperintensity lacks specificity to be clinically useful (Filippi *et al.*, 2010). Post-mortem studies have identified widespread extramotor as well as motor white matter degeneration (Smith, 1960). Advanced applications of MRI have demonstrated patterns of structural and functional change in the brain (and spinal cord) of patients with ALS, but mainly in cross-sectional studies (Turner *et al.*, 2012). Specifically, a core white matter signature for ALS involving the corticospinal tracts and interhemispheric, largely motor callosal fibres, is consistently identified by diffusion tensor imaging (DTI) (Filippini *et al.*, 2010). Meta-analysis of studies using voxel-based morphometry of the segmented grey matter, revealed changes confined to the primary motor cortex in ALS (Chen and Ma, 2010). More widespread grey matter changes have been most prominent in studies of the 10–15% of patients with ALS with frank frontotemporal dementia (Chang *et al.*, 2005), or in ALS genotypes such as *C9orf72*, which are more consistently associated with marked frontotemporal dementia-spectrum cognitive impairments (Bede *et al.*, 2013*a*).

Longitudinal studies in ALS are challenging due to the typically rapid progression of disability and physical demands of MRI scanning. In the small number of studies undertaken to date, significant white and grey matter changes over time have been observed in some (Agosta *et al.*, 2009; Senda *et al.*, 2011; van der Graaff *et al.*, 2011; Keil *et al.*, 2012; Verstraete *et al.*, 2012), but not others (Blain *et al.*, 2007; Mitsumoto *et al.*, 2007; Sage *et al.*, 2007). A study of a small number of patients with ALS scanned longitudinally specifically noted motor cortex grey matter atrophy over time, but no progression in the reduction in fractional anisotropy (a measure of fibre tract integrity) of corticospinal tract white matter (Kwan *et al.*, 2012).

## Materials and methods

### Participants

Prevalent and incident cases of ALS from a large tertiary referral clinic were offered participation in the Oxford Study for Biomarkers in MND (‘BioMOx’) between 2009 and 2013. Sixty patients with sporadic ALS underwent initial (baseline) MRI at enrolment. Initial symptom onset was bulbar (*n* = 12), right-sided arm or leg (*n* = 26), left-sided arm or leg (*n* = 19), both legs (*n* = 1), trunk (*n* = 1) and cognitive impairment (*n* = 1). Thirty-six healthy control subjects were available. Features of participant groups are summarized in Table 1.

**Table 1:** Summary of study participant features

Group	Age (years)	Sex (f:m)	Duration (months)	ALSFRS-R	UMN score	Rate of progression	ACE	Verbal fluency (letter)	Verbal fluency (animals)	Memory recall
ALS, T_0 _(*n = *60)	61 (11)	17:43	37 (30)	35 (6)	9 (4)	0.60 (0.59)	91 (7)	5 (1)	6 (1)	5 (2)
ALS, T_0 _(*n = *27)	59 (12)	10:17	45 (34)	35 (4)	9 (4)	0.47 (0.35)	94 (5)	6 (1)	6 (1)	6 (2)
ALS, T_MAX_ (*n = *27)	61 (12)	10:17	61 (40)	28 (6)	9 (5)	0.51 (0.48)	95 (7)	5 (1)	6 (1)	6 (2)
Δ T_0_ − T_MAX_ (*n = *27)	1 (1)	–	16 (8)	7 (5)	1 (1)	0.09 (0.15)	3 (2)	1 (1)	1 (1)	1 (1)
Controls	53 (13)	16:20	–	–	–	–	98 (3)	7 (0)	7 (1)	6 (1)

UMN = upper motor neuron score (mean total score and subscores reported for subsets of patients with data available).

Ethical approval for all procedures was obtained in advance (South Central Oxford Ethics Committee: 08/H0605/85), with written informed consent obtained from all participants. All patients were notably apparently sporadic i.e. reporting no family history of ALS or frontotemporal dementia. All patients were diagnosed by one of two experienced neurologists (K.T., M.R.T.) according to standard criteria (Brooks *et al.*, 2000). A smaller proportion of these patients have appeared in previously published studies undertaken as the cohort was still being recruited (Filippini *et al.*, 2010; Douaud *et al.*, 2011; Menke *et al.*, 2012; Kolind *et al.*, 2013; Stagg *et al.*, 2013).

Participants underwent clinical examination on the day of study (M.R.T.). The revised ALS Functional Rating Scale (ALSFRS-R) was used to assess disability (including analysis of bulbar, upper and lower limb subscores, with lower total score reflecting higher disability) (Cedarbaum *et al.*, 1999). Disease duration was calculated from symptom onset to scan date in months, and the rate of progression was then determined as: (48 − ALSFRS-R)/disease duration. An upper motor neuron clinical burden score out of 15 comprised the number of pathologically brisk reflexes [extensor plantar responses (2), brisk facial and jaw jerks (3), biceps (2), supinator (2), triceps (2), finger (2), knee (2), and ankle reflexes] (Turner *et al.*, 2004). Cognitive assessment was undertaken using the revised Addenbrooke’s Cognitive Examination Score (ACE-R, maximum score 100, with subscores for verbal fluency for ‘p words in 1 minute’, ‘animals in 1 minute’, and a memory task, each scored 0–7) (Mioshi *et al.*, 2006).

Follow-up MRI was conducted approximately every 6 months, to a maximum of five scans in total, and the last scan (T_MAX_) used in comparison with the first (T_0_). A group of 36 healthy control subjects underwent MRI and cognitive assessment on a single occasion.

### Image acquisition

Scans were performed at the Oxford Centre for Clinical Magnetic Resonance (OCMR) using 3 T Siemens Trio scanner (Siemens AG) with a 12-channel head coil. For each subject T_1_-weighted images were obtained using a 3D MP-RAGE sequence (192 axial slices, flip angle: 8°, 1 × 1 × 1 mm^3^ voxel size, echo time/repetition time/inversion time = 4.7 ms/2040 ms/900 ms). Acquisition time for the MP-RAGE image was 6 min. Whole-brain DTI images were acquired using an echoplanar imaging sequence (60 isotropic directions; b-value = 1000 s/mm^2^; echo time/repetition time = 94 ms/10 000 ms; 2 × 2 × 2 mm^3^ voxel size; 65 slices). In addition, four images without diffusion weighting were acquired. Furthermore, a field map was acquired using a gradient echo imaging sequence (2 × 2 × 2 mm^3^ voxel size; 65 slices; echo time 1/echo time 2/repetition time = 5.19 ms/7.65 ms/655 ms) to account for distortions caused by field inhomogeneities.

### Image analysis

All images were analysed using tools from the FMRIB Software Library (FSL) (Smith *et al.*, 2004). Note that DTI data (a number of scans had to be excluded due to artefacts in the raw data) and that ACE-R total and subscores were not available for every subject. Furthermore, not all patients included in this study had a follow-up scan (see flow chart for participants in Supplementary Fig. 1).

### Voxel-based morphometry

T_1_-weighted MP-RAGE data was analysed with FSL-VBM, a voxel-based morphometry (VBM) style analysis (Good *et al.*, 2001; Smith *et al.*, 2004; Douaud *et al.*, 2007). For group comparison, a standard optimized FSL-VBM protocol was run for the structural images for all 36 control subjects and for structural images for the first time point for 36 age-matched patients (T_0_). First, structural images were brain-extracted (Smith, 2002). Next, tissue-type segmentation was carried out using FAST4 (Zhang *et al.*, 2001). The resulting grey matter partial volume images were then aligned to MNI152 standard space using the affine registration tool FLIRT (Jenkinson and Smith, 2001; Jenkinson *et al.*, 2002), followed by non-linear registration using FNIRT (Andersson *et al.*, 2007). The resulting images were averaged to create a symmetric, study-specific template, to which the native grey matter images were then non-linearly re-registered. We then multiplied the registered partial volume images of all subjects by the Jacobian of the warp field (‘modulation’) to correct for local expansion or contraction. The modulated segmented images were then smoothed with an isotropic Gaussian kernel with a sigma of 3 mm.

Furthermore, standard optimized FSL-VBM protocol processing as described above was run separately in the patient group only for all 60 patients’ structural images at the first time point (T_0_) in preparation for subsequent correlational analyses.

As part of the preprocessing pipeline for the FSL-VBM longitudinal analysis, for each of the 27 patients who had both structural images and DTI images for at least two time points (T_0_ and T_MAX_), images were bias field-corrected, brain-extracted, and halfway linear registration matrices between T_0_ and T_MAX_ scans were calculated. For all subjects, the bias field corrected T_0_ and T_MAX_ images in native space were then linearly registered into halfway space and averaged. Subsequently, using FNIRT, the bias field corrected T_0_ and T_MAX_ images were non-linearly registered to their average image in halfway space that was obtained after linear registration.

In parallel, tissue-type segmentation was carried out in native T_1_-weighted space using FAST4 for all bias field corrected and brain-extracted T_0_ and T_MAX_ scans. The resulting grey matter partial volume images were then aligned into MNI space using the affine registration tool FLIRT, followed by non-linear registration using FNIRT. The resulting images were averaged to create a study-specific grey matter ‘longitudinal’ template in MNI space.

Subsequently, the grey matter images in native T_1_-weighted space for both time points were non-linearly registered into halfway space, and their average in halfway space was then non-linearly registered to this study-specific template. Finally, a combination of the previously described transformations was used to register the native T_0_ and T_MAX_ grey matter images to MNI space. We then multiplied the registered partial volume images of all subjects by the Jacobian resulting from the combination of the two warp fields (‘modulation’) to correct for local expansion or contraction. The modulated segmented images were then smoothed with an isotropic Gaussian kernel with a sigma of 3 mm.

### Diffusion tensor imaging general preprocessing

Each subject’s DTI scans were corrected for head motion and eddy currents and then brain-extracted to remove any non-brain voxels. To correct for B_0_ inhomogeneities and unwarp scans, field map correction was performed with FUGUE. Fractional anisotropy, mean diffusivity, axial diffusivity (eigenvector L1) and L2 and L3 maps were created using DTIFIT by applying a diffusion tensor model to each voxel (Behrens *et al.*, 2003). Radial diffusivity maps were created by averaging the L2 and L3 maps [radial diffusivity = (L2 + L3)/2].

#### Diffusion tensor imaging tract-based spatial statistics preprocessing

For time point T_0_, all individual fractional anisotropy images of all subjects (data from 22 controls and 39 patients for the preprocessing for the group comparison, 43 T_0_ images for the preprocessing for correlational analyses in patients) were non-linearly registered to a standard fractional anisotropy template (http://fsl.fmrib.ox.ac.uk/fsl/fslwiki/FMRIB58_FA), and then averaged to create a study-specific template to which each subject’s fractional anisotropy map was then non-linearly registered. Next, the mean fractional anisotropy image was created and thinned to create a mean fractional anisotropy skeleton (Smith *et al.*, 2006), which represents the centres of all tracts common to the group. Each subject’s aligned fractional anisotropy data were then projected onto this skeleton. The same operations that were used to register the individual fractional anisotropy images to the study-specific template and project fractional anisotropy values onto the mean fractional anisotropy skeleton were subsequently applied to the individual mean diffusivity, L1, and radial diffusivity images.

#### Longitudinal diffusion tensor imaging data preprocessing

As there is no optimized implementation for preprocessing longitudinal data available within tract-based spatial statistics (TBSS), we non-linearly registered the individual fractional anisotropy images for both time points (*n = *27) to the respective T_1_-weighted images and combined this transformation with the transformation that was used to register the native T_0_ and T_MAX_ grey matter images to MNI space. The combination of these two transformations was applied to fractional anisotropy, mean diffusivity, L1, and radial diffusivity maps in native space for both time points to register them into MNI space.

### Statistical analyses

The Juelich Histological Atlas was used to produce masks of the left and right primary motor cortex and frontal lobe for VBM analysis, and left and right corticospinal tract, superior longitudinal fascicle, and callosal body (corpus callosum) for TBSS analysis in MNI standard space. Primary motor cortex and corticospinal tract are obvious region of interest choices in ALS. Corpus callosum and superior longitudinal fascicle regions of interest were chosen based on previously published studies reporting involvement of extra-motor tracts in ALS (Filippini *et al.*, 2010; Sarro *et al.*, 2011; Trojsi *et al.*, 2013). For the TBSS analysis, where the final region of interest resulted from the intersection of the atlas-based masks and the mean fractional anisotropy skeleton mask, left and right corticospinal tract and corpus callosum masks were thresholded at ‘45’ and the left and right superior longitudinal fascicle masks were thresholded at ‘10’.

Clinical and cognitive scores where available [upper motor neuron score, ALSFRS-R total score and subscores, and progression rate (VBM: *n = *60, TBSS: *n = *43), and ACE total (VBM: *n = *32, TBSS: *n = *22) and subscores (VBM: *n = *45, TBSS: *n = *35)] were then regressed with the preprocessed VBM and TBSS images (for the respective T_0_ MRI scans) (Supplementary Fig. 1).

To assess clinical correlations (using age as covariate of no interest) and group differences (‘two groups, unpaired’ test as implemented in FSL) between controls (VBM: *n = *36, TBSS: *n = *22) and age-matched patients (VBM: *n = *36, TBSS: *n = *39), and longitudinal grey and white matter decline between T_0_ and T_MAX_ (‘two groups, paired’ test as implemented in FSL; *n = *27), voxel-wise general linear model was applied using permutation-based non-parametric testing. Results were considered significant for *P < *0.05, after correction for multiple comparisons (family-wise error, FWE) within each region of interest (whole brain as well as masks derived from the Juelich Histological Atlas), using the threshold-free cluster enhancement (TFCE) approach (Smith and Nichols, 2009).

## Results

### Participants

To achieve age matching between groups, structural scans (T_0_) of 36 patients (54.2 ± 8.6 years) were selected from the available 60 scans for the VBM comparison with structural data of all 36 controls (*P = *0.652). Similarly, for the respective TBSS analysis, available DTI data from 22 control subjects (56.2 ± 10.1 years) were compared with data from 39 selected patients (59.0 ± 10.2 years; *P = *0.303).

### Magnetic resonance imaging group comparison and longitudinal analysis

**Figure 1:**
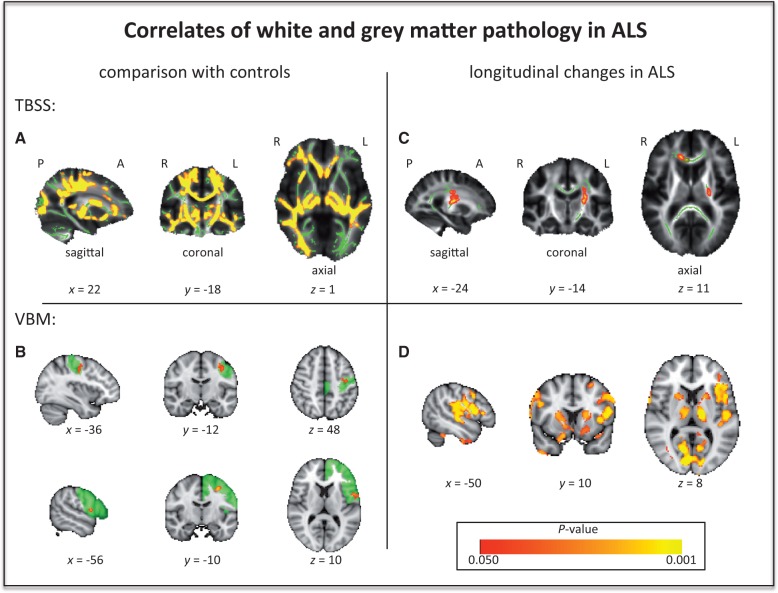
Significant (*P < *0.05, family-wise error corrected) fractional anisotropy (**A**) and grey matter (**B**) differences between patients and control subjects. Significant (*P < *0.05, FWE corrected) longitudinal diffusivity increases (**C**) and grey matter loss (**D**) in patients. TBSS results are overlaid onto the group’s mean fractional anisotropy skeleton (green, for region of interest analyses only the intersection of the skeleton with the respective region of interest is shown) and the group’s mean fractional anisotropy image (greyscale). VBM results are overlaid onto the MNI T_1_ (2 mm) template (left primary motor cortex and frontal lobe regions of interest are outlined in green where applicable). P = posterior; A = anterior; R = right; L = left; *x*, *y*, *z* = coordinates in MNI space.

Examining the whole TBSS skeleton, significantly lower fractional anisotropy (Fig. 1A) and co-localized higher mean diffusivity, axial diffusivity (L1) and radial diffusivity was observed in widespread white matter regions in the patient group at first time point T_0_. VBM analysis demonstrated significantly lower grey matter volume in patients at baseline only in the left primary motor cortex region of interest and in the left frontal lobe region of interest (Fig. 1B).

Longitudinal DTI revealed localized significant increases for L1 and mean diffusivity in the corpus callosum region of interest, and increases for L1 in the left corticospinal tract region of interest (Fig. 1C). The longitudinal VBM analysis showed grey matter volume decreases encompassing widespread areas including motor and frontotemporal regions, and the thalami and caudate heads bilaterally (Fig. 1D).

### Upper motor neuron score

**Figure 2:**
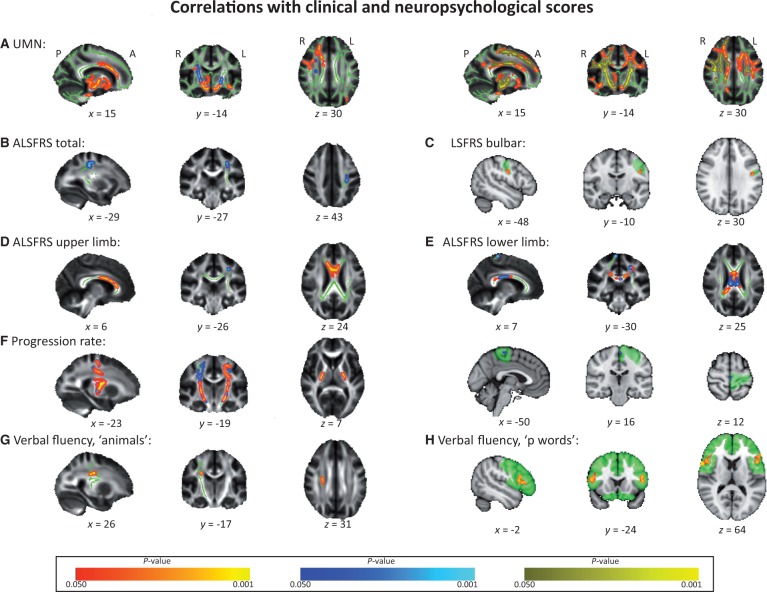
Results of significant (*P < *0.05, family-wise error corrected) grey matter (VBM) and white matter microstructure (TBSS) correlations with clinical and neuropsychological scores in the patient cohort. TBSS results are overlaid onto the group’s mean fractional anisotropy skeleton (green; for region of interest analyses only the intersection of the skeleton with the respective region of interest is shown) and the group’s mean fractional anisotropy image (greyscale). VBM results are overlaid onto the MNI T_1_ (2 mm) template; respective regions of interest are outlined in green. Positive correlations are displayed in red/yellow colour; negative correlations are shown in blue–light blue colour. (**A**) *Left*: fractional anisotropy = blue, L1 = red–yellow; *Right*: mean diffusivity = red–yellow, radial diffusivity = yellow. (**D**) Fractional anisotropy = red–yellow, radial diffusivity = blue. (**E**) Fractional anisotropy = red–yellow, radial diffusivity/mean diffusivity/L1 = blue. (**F**) Fractional anisotropy = blue, radial diffusivity = red–yellow. UMN = upper motor neuron score; P = posterior; A = anterior; R = right; L = left; *x*, *y*, *z* = coordinates in MNI space.

The mean baseline upper motor neuron score for all patients was 9 ± 4 (range 0–15). Examining the whole white matter TBSS skeleton, higher upper motor neuron scores were associated with a significant decrease of fractional anisotropy seen mainly in the corticospinal tracts with co-localized increases in L1 (Fig. 2A), and an increase of mean diffusivity in both the corticospinal tracts, superior longitudinal fascicles, and in the corpus callosum, with co-localized increases in radial diffusivity (Fig. 2A). Whole-brain voxel-wise VBM analysis did not yield any significant correlations with the upper motor neuron score.

The region of interest approach furthermore revealed significantly lower fractional anisotropy in the right superior longitudinal fascicle.

There was notably limited variability in individual patient upper motor neuron scores over time (Supplementary Fig. 2A).

### ALSFRS-R total score and progression rate

The mean baseline ALSFRS-R score for all patients was 35 ± 6 (range 18–44). Neither whole-brain voxel-wise TBSS analyses, nor VBM analysis revealed any significant correlations with the ALSFRS-R total score. Region of interest analysis on the other hand demonstrated a negative correlation between radial diffusivity and total ALSFRS-R score in the region of the left superior longitudinal fascicle (where the superior longitudinal fascicle region of interest overlaps with the corticospinal tract region of interest) (Fig. 2B). There were no significant VBM correlations in any of the regions of interest.

There was no significant correlation with progression rate in the whole-brain TBSS or VBM analyses. Region of interest analysis showed that a higher baseline progression rate was associated with significantly lower fractional anisotropy and significantly higher radial diffusivity in the left and right corticospinal tract (Fig. 2F), significantly lower fractional anisotropy in the right superior longitudinal fascicle, and significantly higher L1 in the corpus callosum. The VBM analysis did reveal a significant negative correlation between progression rate and grey matter volume in the left primary motor cortex region of interest (Fig. 2F).

In contrast to the change in individual ALSFRS-R scores between assessments (Supplementary Fig. 2B), there was notably limited individual variability across a range of progression rates (Supplementary Fig. 2C).

### ALSFRS-R regional subscores

VBM volume within a grey matter region compatible with the bulbar segment of the left primary motor cortex was positively correlated with bulbar disability subscore (Fig. 2C).

VBM analysis did not reveal any significant correlation for the upper limb subscore. Radial diffusivity correlated negatively with upper limb disability subscore for a small region in the left superior longitudinal fasciculus in the region of interest analysis. Furthermore, region of interest analysis revealed a significant positive correlation for fractional anisotropy in the corpus callosum (Fig. 2D).

In the region of interest analyses, the lower limb disability score correlated negatively with VBM measures in the left primary motor cortex region of interest. Furthermore, lower limb scores were positively correlated with fractional anisotropy values in the callosal body and the left superior longitudinal fascicle mask, with co-localized negative correlation for radial diffusivity in both structures, and mean diffusivity in the left superior longitudinal fascicle mask (where superior longitudinal fascicle and corticospinal tract mask overlap). The score furthermore correlated negatively with mean diffusivity in the right corticospinal tract (co-localized with negative correlation with L1) (Fig. 2E).

### Cognitive assessments

Total ACE score was not correlated with any significant grey or white matter pathology. Region of interest TBSS analysis revealed a positive correlation for verbal fluency for ‘animals’ and L1 in a small region in the right corticospinal tract (Fig. 2G). Region of interest VBM analysis using a frontal lobe mask demonstrated a significant positive correlation for grey matter volume and verbal fluency for ‘p’ words in Broca’s area and, its homologue and the dorsolateral prefrontal region (dorsolateral prefrontal cortex) bilaterally (Fig. 2H), but no correlations for ‘animals’ or memory task.

## Discussion

The key findings of this longitudinal study may be summarized as follows. First, VBM-based grey matter abnormalities in apparently sporadic ALS evolves longitudinally, encompassing clinically silent areas, including the basal ganglia. Second, the core cerebral white matter involvement, mainly involving the corticospinal tracts, is linked more strongly to clinical upper motor neuron involvement rather than disability score as measured by the ALSFRS-R. Third, this core white matter DTI signature does not seem to significantly evolve, in keeping with the novel observation that individual clinical upper motor neuron scores show rather limited variability as disability proceeds. Fourth, limited topographically-appropriate grey and white matter correlates for regional disability are identifiable. Finally, the involvement of Broca’s area and dorsolateral prefrontal cortex in relation to verbal fluency impairment is specifically confirmed.

The clinical heterogeneity of ALS is well recognized, and the condition may be best considered a syndrome. Less than 10% of all cases of ALS are associated with hexanucleotide expansions in *C9orf72*, or pathological mutations in *SOD1*, *TARDBP* or *FUS*. Moreover, such cases are clinically indistinguishable from apparently sporadic cases, and encompass the full range of clinical phenotypes. The conclusion is that there may be several upstream points of access to a neurodegenerative process (Al-Chalabi and Hardiman, 2013; Turner *et al.*, 2013), one that then consistently affects upper, lower and extramotor neurons, but to varying extents, typically with the common endpoint of respiratory failure.

### Magnetic resonance imaging studies in relation to phenotype

The consistent DTI changes identified previously within the corticospinal tracts and corpus callosum (Kassubek *et al.*, 2005; Filippini *et al.*, 2010) have been replicated in many studies, and also related to clinical upper motor neuron signs (studies comprehensively reviewed in Turner *et al.*, 2012). Upper motor neuron involvement is demonstrable pathologically even in ALS cases with only lower motor neuron involvement clinically (Brownell *et al.*, 1970; Ince *et al.*, 2003), and its detection alongside the more typical lower motor neuron involvement in ALS is a cornerstone of diagnostic certainty. Corpus callosum changes are mainly located in the interhemispheric motor fibres of the body (Chapman *et al.*, 2013), and seem exaggerated in patients with primary lateral sclerosis which are considered part of the spectrum of ALS in the wider use of the diagnostic term (Ciccarelli *et al.*, 2009; Agosta *et al.*, 2013).

Clinicopathological correlations in ALS suggest a pathological process involving, at least in part, a top–down cortical degeneration topographically related to regional anterior horn involvement (Eisen *et al.*, 1992; Ravits and La Spada, 2009; Braak *et al.*, 2013). A previous VBM study demonstrated specific correlation of cortical volume within topographically-appropriate regions of the primary motor cortex for bulbar and upper limb disability subscores as well as overall ALSFRS-R (Bede *et al.*, 2013*b*). Some DTI studies have noted a positive correlation for fractional anisotropy and total ALSFRS-R (Thivard *et al.*, 2007; Keil *et al.*, 2012). The present study provides further support for this concept in relation to bulbar and lower limb disability.

Advanced MRI studies in relation to cognitive impairments support the now well-established clinical, pathological and genetic links between ALS and frontotemporal dementia. Marked frontotemporal grey matter changes have been observed in those with frank frontotemporal dementia (Chang *et al.*, 2005), and in those ALS cases linked to *C9orf72* hexanucleotide repeat expansions, known to have consistent cognitive impairments (Byrne *et al.*, 2012). Indeed it has been suggested that extramotor cortical changes observed in ALS studies conducted before the discovery of this genetic locus, were perhaps driven by the inclusion of such individuals (Bede *et al.*, 2013*a*), though our study of apparently sporadic patients suggests that these grey matter changes may be common to all cases of ALS eventually, rather than being genotype-specific, including those changes in the basal ganglia, also noted by others (Bede *et al.*, 2013*c*; Fekete *et al.*, 2013; Sharma *et al.*, 2013).

Specific cognitive impairments in patients with ALS have been studied regionally in relation to DTI metrics, with attention and executive dysfunction linked to the corpus callosum, corticospinal tract, cingulum, inferior longitudinal, inferior fronto-occipital, and uncinate fasciculi; verbal learning and memory test scores to the fornix; and visual-spatial abilities to the left uncinate fasciculus (Sarro *et al.*, 2011). Verbal fluency is recognized as one of the most consistent impairments across a range of ALS phenotypes without overt cognitive dysfunction (Abrahams *et al.*, 2000). Activation studies have linked verbal fluency to Broca’s area and the dorsolateral prefrontal cortices in healthy individuals (Schlosser *et al.*, 1998), confirmed using activation PET in patients with ALS (Abrahams *et al.*, 1996), and with evidence of specific regional microglial activation (Turner *et al.*, 2004). Modified T_2_-weighted sequences sensitive to myelin loss have also demonstrated selective involvement of the projection fibres of the anterior corpus callosum in relation to impaired verbal fluency in ALS (Kolind *et al.*, 2013). The present study confirms the importance of the dorsolateral prefrontal cortex region in relation to VBM-based grey matter volume change linked to verbal fluency impairment.

### Implications for magnetic resonance imaging-based biomarkers and timing of cerebral pathology in amyotrophic lateral sclerosis

The biomarker needs in ALS are multiple and distinct (Bowser *et al.*, 2011), and MRI offers significant potential in all regards (Turner *et al.*, 2011). The majority of cases of ALS are clinically obvious upon presentation to a neurologist, but are currently delayed in their referral by lack of early recognition of symptoms by patient and primary care physician, or inappropriate referral to non-neurological specialists. The major value in an objective diagnostic biomarker for ALS would be in distinguishing lower motor neuron-predominant cases that typically have among the longest diagnostic delay, and may be otherwise excluded from clinical trials. The ability of MRI to detect clinically occult upper motor neuron involvement has obvious potential. Our study confirms that the rostral corticospinal tract DTI signature is the leading diagnostic MRI candidate at present. The priority in terms of experimental work must now be to test these emerging candidates in disease control populations, rather than alongside healthy control subjects, ideally those that mimic lower motor neuron-predominant forms of ALS such as multifocal motor neuropathy with conduction block, or that may mimic primary lateral sclerosis, such as primary progressive multiple sclerosis.

The recognition of significant clinical heterogeneity in ALS, despite a common pathological signature of cytoplasmic TARDBP inclusions in nearly all cases, suggests that there may ultimately be a range of biomarker signatures linked to specific phenotypes or progression rates. We observed some regional grey and white matter changes in relation to clinical features, and in exploratory region of interest analysis confirmed our previous observations of correlation of fractional anisotropy within the corticospinal tract in relation to rate of progression (Menke *et al.*, 2012). However the goal of translating MRI-based metrics for the stratification of patients with ALS, for example in the context of a clinical trial, does not seem likely at present in comparison with more easily acquired examination-based information. Studies of baseline MRI measures in relation to overall survival (Agosta *et al.*, 2010), or surface-based morphometry change in the temporal lobes (Verstraete *et al.*, 2012), may offer further potential in this regard. The integration of functional MRI markers may prove to be crucial for achieving the necessary biomarker sensitivity and specificity (Douaud *et al.*, 2011).

In contrast to the observed longitudinal widespread grey matter changes, we only found few significant changes in the white matter metrics over time (which furthermore only occur in regions that overlapped with areas in which we found positive correlations of T_0_ mean diffusivity and L1 values with age; data not shown). Although the sensitivity, limitations and potential confounds of DTI are well recognized, the additional observation of a lack of change for most patients in their clinical upper motor neuron score over time (not previously reported to our knowledge) may have implications for the sequence of pathological change in ALS, and understanding the variable regional spread of symptoms. We suggest that the upper motor neuron lesion in ALS is not only variable among individuals but may be relatively constant, in clinical terms at least, in the symptomatic period. This needs to be validated in another population. Within a similar theme, it has been noted that cognitive involvement does not generally progress significantly in those without major impairments at baseline (Elamin *et al.*, 2013), supporting a broader concept of variably ‘compartmentalized’ symptoms in ALS (between upper motor neuron, lower motor neuron and frontotemporal neuronal populations). Understanding the anatomical or physiological substrate for any such limits in involvement may be the essential clue to effective disease-modifying therapies.

Meanwhile, there are important implications for the development of biomarkers sensitive to therapeutic intervention if future studies support the relative lack of white matter change as detectable by TBSS over time. Our demonstration of widespread, ‘progressive’ grey matter involvement over time suggests that cortically-based measures may be the most suitable in this regard. One limitation of our study is the lack of follow-up data in the control group that would help to distinguish between changes that occur due to ageing and disease-related changes over time. However, because of the relatively short time interval between the two scans (on average <16 months) and the fact that the longitudinal VBM results observed in our patient group remain qualitatively unchanged after adding the time interval between T_0_ and T_MAX_ scan as covariate of no interest and furthermore did not overlap with areas in which we found negative correlations with age in a cross-sectional analysis using the respective T_0_ T_1_-weighted data (data not shown), we believe the observed longitudinal changes are unlikely to be caused by ageing alone. We believe that white matter pathology is likely to be established significantly before the onset of symptoms in ALS, although it is acknowledged that disease duration at first scan in our study was an average of 2–3 years after symptom onset. Asymptomatic carriers of pathogenic mutations in *SOD1* were reported to have detectable fractional anisotropy decreases in the corticospinal tracts (Ng *et al.*, 2008), and further DTI studies in similar patient groups are underway (Benatar and Wuu, 2012).

In summary, ALS may be characterized initially by a predominantly white matter tract pathological signature, evolving as a widespread cortical network degeneration over time.

## Supplementary Material

Supplementary Data

## Abbreviations

ACE-R: Addenbrooke’s Cognitive Examination Score-Revised
ALS: amyotrophic lateral sclerosis
ALSFRS-R: ALS Functional Rating Scale-Revised
DTI: diffusion tensor imaging
TBSS: tract-based spatial statistics
VBM: voxel-based morphometry
