# Supplementary material for: Widespread grey matter pathology dominates the longitudinal cerebral MRI and clinical landscape of amyotrophic lateral sclerosis
Source: Brain. 2014 Jun 20;137(9):2546–55. doi: 10.1093/brain/awu162 (PMC4132644; doi:10.1093/brain/awu162)
Supplement: Supplementary Data [file supp_137_9_2546__index.html]

Widespread grey matter pathology dominates the longitudinal cerebral MRI and clinical landscape of amyotrophic lateral sclerosis — Supplementary Data 

# Widespread grey matter pathology dominates the longitudinal cerebral MRI and clinical landscape of amyotrophic lateral sclerosis

## Supplementary Data

files

**Files in this Data Supplement:**

- Supplementary Data - doc file
