# Supplementary material for: Widespread grey matter pathology dominates the longitudinal cerebral MRI and clinical landscape of amyotrophic lateral sclerosis
Source: Brain. 2014 Jun 20;137(9):2546–55. doi: 10.1093/brain/awu162 (PMC4132644; doi:10.1093/brain/awu162)
Supplement: Supplementary Data [file supp_awu162_Supplementary_Figures_and_Legends.doc]

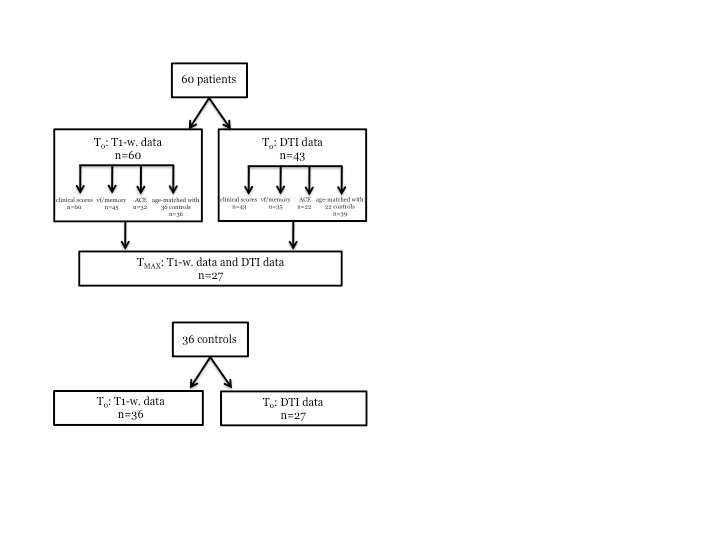


Supplementary figure 1: Flow chart illustrating the number of subjects used for the different statistical analyses. For both time points, a proportion of DTI scans had to be discarded prior to processing due to artefacts. Clinical and neuropsychological scores available for correlations with MRI data included ALSFRS-R (total and sub-scores), UMN score, and progression rate, and total ACE scores, as well as sub-scores for verbal fluency (‘p words’ and ‘animals’) and memory.


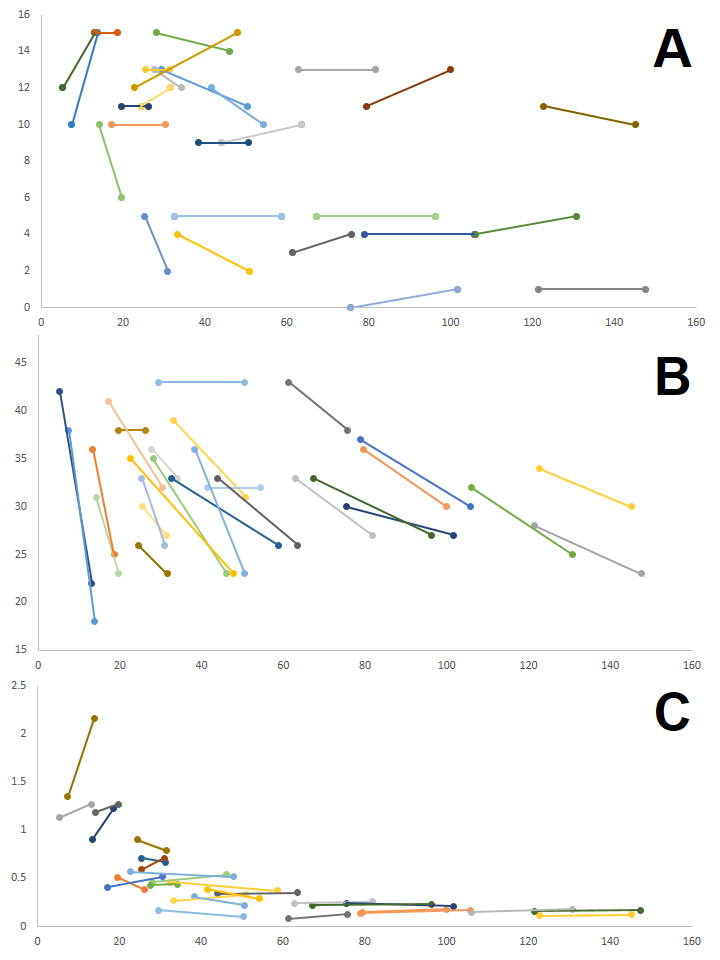


Supplementary figure 2: Changes in clinical scores of ALS patients during the longitudinal study. UMN score (A), ALSFRS-R (B) and progression rate (C). UMN scores and rates of progression were generally constant for individuals in established disease.
